# Supplementary material for: Patterns of Intron Gain and Loss in Fungi
Source: PLoS Biol. 2004 Nov 30;2(12):e422. doi: 10.1371/journal.pbio.0020422 (PMC532390; doi:10.1371/journal.pbio.0020422)
Supplement: Table S1 — Also available at http://genes.mit.edu/NielsenEtAl/. (4.3 MB ZIP). [file pbio.0020422.st001.zip › NielsenEtAl/html/77.html]

AN5785.1.NCU07956.1.MG05136.1.FG01345.1


```
 CLUSTAL W (1.82) Multiple Sequence Alignments - Introns Inserted


Sequence 1: NCU07956.1	257 aa
Sequence 2: FG01345.1	255 aa
Sequence 3: AN5785.1	258 aa
Sequence 4: MG05136.1	264 aa
Alignment Length: 267 aa
Number Identitical Residues: 110 aa
Alignment Score (without introns) 5596


MG05136.1 	MPAPTAVKPTAAIEDGAAVAPVADNGSMAVEAD~QAEEFMLDTTDQTLPAASDIAAPTEE
NCU07956.1	MPAPTALR-------QPVKAPAA--QIPIQTE-~QEDELILDVNEAVNLTDPSAAAPPSN
FG01345.1 	MPAPTALK-------QAESAPLAN-DISLPVEG1ADEEFLLDAPGAED-ADANVLVPVED
AN5785.1  	MPAPTALR-------AASDASTTETPIAPHAQE1HDEEILIDVQPSNNADDVLASANSAE
          	******::        .  *. :..           :*:::*.            .   :

MG05136.1 	NTE--MAVDEESRPKFSAAKDE~GPSVMKETRKVPIPPHRMTPLKKDWTQI~YEALVEHL
NCU07956.1	DQDVQMAVDEEGRPRFAPGQDV~QIIRRAETRKIGIPPNRMSALKANWTKI2YPPLVEHC
FG01345.1 	SND--MQIDEEGRPRFAPARDI0DPVTRVETRKIPIPPHRMTPLKQSWTSI~YPPLVEHL
AN5785.1  	DTD--MRIDLEGRPVFTPAKDT~PAAYRVETRKVPVPPHRMTPLKANWPKI~YPPLVEHL
          	. :  * :* *.** *:..:*        ****: :**:**:.** .*..* * .**** 

MG05136.1 	KLQV2RVNTRTKAVELRTSEHTTEAGSLQKGE1DFVKAYTLGFEVQDAIALIRVPELYIQ
NCU07956.1	KLQV~RMNIKEKRVELRSSRHTTTPESLQMGA~DFVSAFAMGFDIDDAIALLRLDSLYIQ
FG01345.1 	KLQC~RMNIKRKTVELRSSKHTTESGALQKGE~DFVKAFTLGFDVDDAIALLRLDDLYIQ
AN5785.1  	KLQV~RINIKSRAVELRTSKFTTDTGALQKGE~DFVKAFTLGFDIDDAIALLRLDDLYIR
          	***  *:* : : ****:*..** . :** *  ***.*:::**:::*****:*: .***:

MG05136.1 	TFEI1KDVKSLTG-DHLARGVGRIAGKDGKTKFAIENA~SRTRIVLADSK0IHILGSFRN
NCU07956.1	SFDV~KDVRQTLGPDALGRAIGRIAGKDGKTKFAIENA1TKTRIVLAGSR~VHILGAFEN
FG01345.1 	SFEI~KDVRTMHG-DSQARAIGRIAGKDGKTKFAIENA~SRTRIVLADSK~IHILGGFKN
AN5785.1  	SFEI~RDVKASLNGEHLSRAIGRIAGKDGRTRHAIENA~SRTRIVIADQK~IHILGRFQN
          	:*:: :**:   . :  .*.:********:*:.***** ::****:*..: :**** *.*

MG05136.1 	VRMAQESIVDLILGKPP~SKVYGNLRTISARQKERF
NCU07956.1	IGMARESVVSLILGHQP~GKVYNNLRIIASRQKERF
FG01345.1 	IHLARESVVSLILGKPP~GKVYGNLRTVAARMKERF
AN5785.1  	INAGQEAVVSLILGAPP0GKVYGNLRKVAARMKERF
          	:  .:*::*.****  * .***.*** :::* ****
```
